# Spatial distribution, diversity, and taphonomy of clypeasteroid and spatangoid echinoids of the central Florida Keys

**DOI:** 10.7717/peerj.14245

**Published:** 2022-10-31

**Authors:** Tobias B. Grun, Michał Kowalewski

**Affiliations:** Florida Museum of Natural History, University of Florida, Gainesville, Florida, United States of America

**Keywords:** Sea urchin, Ecology, Life-dead fidelity, Bio-inventory, Habitat comparison, Echinodermata

## Abstract

**Background:**

Irregular echinoids are ecosystem engineers with diverse functional services. Documenting present-day distribution of those widespread organisms is important for understanding their ecological significance and enhancing our ability to interpret their rich fossil record.

**Methods:**

This study summarizes SCUBA surveys of clypeasteroid and spatangoid echinoids conducted in 2020 and 2021 along the central part of the Florida Keys. The survey included observations on both live and dead specimens, their distribution, habitat preferences, abundance, and live-dead comparison.

**Results:**

Echinoids were found at 17 out of 27 examined sites (63%) and occurred across a wide range of habitats including coastal seagrass meadows, subtidal sand and seagrass settings of the Hawk Channel, backreef sands, and fine muddy sands of deeper forereef habitats. The encountered species, both dead and alive, included *Clypeaster rosaceus* (four sites), *Clypeaster subdepressus* (five sites), *Encope michelini* (three sites), *Leodia sexiesperforata* (eight sites), *Meoma ventricosa* (nine sites), and *Plagiobrissus grandis* (four sites). All sites were dominated by one species, but some sites included up to five echinoid species. Live-dead fidelity was high, including a good agreement in species composition of living and dead assemblages, congruence in species rank abundance, and overlapping spatial distribution patterns. This high fidelity may either reflect long-term persistence of local echinoid populations or fragility of echinoid tests that could prevent post-mortem transport and the formation of time-averaged death assemblages. Regardless of causative factors, the live-dead comparisons suggest that irregular echinoid assemblages, from settings that are comparable to the study area, may provide a fossil record with a high spatial and compositional fidelity. The survey of live fauna is consistent with past regional surveys in terms of identity of observed species, their rank abundance, and their spatial distribution patterns. The results suggest that despite increasingly frequent hurricanes, active seasonal fisheries, massive tourism, and urban development, irregular echinoids continue to thrive across a wide range of habitats where they provide diverse ecosystem services by oxygenating sediments, recycling organic matter, supporting commensal organisms, and providing food to predators. Results reported here document the present-day status of local echinoid populations and should serve as a useful reference point for assessing future regional changes in echinoid distribution and abundance.

## Introduction

Echinoids are one of the unique groups of organisms that are not only ecologically important in many present-day marine ecosystems (*e.g*., Kier & Grant, 1965; Scheibling, 1984; Nebelsick, 2020; Plee & Pomory, 2020 and literature cited therein), but also widespread and diverse in the fossil record (*e.g*., Kroh, 2010; Osborn, Portell & Mooi, 2020). Echinoids are also a prominent member of the Modern Evolutionary Fauna that has dominated marine ecosystems since the late Mesozoic Era (*e.g*., Sepkoski, 1984; Alroy, 2010; Rojas et al., 2021). Consequently, research on present-day echinoids not only benefits our understanding of their distribution and ecological importance today (*e.g*., Weihe & Gray, 1968; Aller & Dodge, 1974; Bell & Frey, 1969; Findlay & White, 1983; Krantzberg, 1985; Reidenauer, 1989; Dahlgren, Posey & Hulbert, 1999; Yeo, Keesing & van Keulen, 2013; Brustolin et al., 2014; Nebelsick, 2020), but can also improve our ability to interpret their rich fossil record (*e.g*., Kidwell & Baumiller, 1990; Nebelsick, 1998; Nebelsick & Kowalewski, 1999; Kroh & Nebelsick, 2003, 2010; Grun, Sievers & Nebelsick, 2014; Grun, Kroh & Nebelsick, 2017; Grun et al., 2018; Mooi, Martínez & Parma, 2016; Grun, 2017; Tyler et al., 2018).

**Figure 1:**
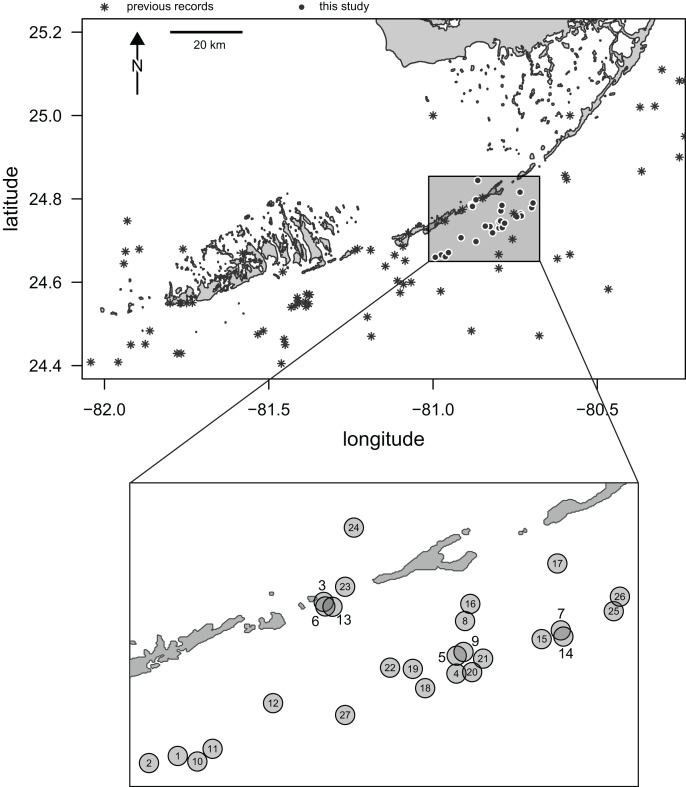
Map of Florida Keys. Study area marked by a rectangle and the sampling sites surveyed in this study indicated by black dots with white outlines. Asterisks indicate archived records of occurrences of irregular echinoids in the region as reported in databases aggregated by the iDigBio portal (search conducted on 10/25/2021). Inset: a close-up of the study area with sites indicated by numbered gray dots. Numbers correspond to site numbers in Table 1 and Appendix 1.

Here, we report the results of a recent survey of live populations and dead remains of irregular echinoids inhabiting the central part of the Florida Keys. Multiple genera of the orders Clypeasteroida and Spatangoida were previously reported from the region in case studies (*e.g*., Kier & Grant, 1965; Chesher, 1969) and additional collecting efforts have been documented in online databases (see details below). However, the existing data are limited in terms of both spatial and temporal coverage. In addition, only a few sampling events have taken place in the last few decades and the central part of the Florida Keys has been particularly poorly sampled (Fig. 1).

Multiple goals motivate this study. First, this is a bio-inventorying effort aimed at assessing the distribution and ecological importance of irregular echinoids in the study region. Second, the study aims to integrate behavioral, ecological, taphonomic, and sedimentological observations to inform our neontological and paleontological knowledge of a group of marine benthic organisms, which is of significant ecological and paleontological importance. Finally, the study includes a joint survey of living populations and dead remains, a comparative approach aimed at evaluating live-dead congruence of echinoid assemblages with paleontological and bio-inventorying implications.

## Materials and Methods

Three SCUBA surveys (August 2020, January 2021, and April 2021) were conducted during daytime along the central Florida Keys in the Long Key area (Fig. 1) by a team of two divers (TBG and MK). A total of 27 sites were surveyed for clypeasteroid and spatangoid echinoids (Fig. 1; Table 1). All surveying and collecting activities were carried out within the scope of the collecting permits #SAL-19-2195-SR and #SAL-18-1294A-SR issued by the Florida Fish and Wildlife Conservation Commission.

**Table 1:** Ordinal rank abundance for the sampled sites estimated separately for live and dead specimens.

Site	Depth	GPS data		*Clypeaster rosaceous*		*Clypeaster subdepressus*		*Encope michelini*		*Leodia sexiesperforata*		*Meoma ventricosa*		*Plagiobrissus grandis*		Total	
	(m)	Latitude	Longitude	Live	Dead	Live	Dead	Live	Dead	Live	Dead	Live	Dead	Live	Dead	Live	Dead
1	22.9	24.6655	−80.9751	0	0	0	+	0	0	0	0	+	+	0	0	x	x
2	13.1	24.6599	−80.9932	0	0	0	0	0	0	0	0	++	0	0	0	x	0
3	1.8	24.7861	−80.8829	0	0	0	0	0	+	+	+	0	0	0	0	x	x
4	27.7	24.7300	−80.7990	0	0	0	0	0	0	0	0	0	0	0	0	0	0
5	8.5	24.7440	−80.7988	0	0	+	0	0	+	+++	+	0	0	+	0	x	x
6	2.1	24.7844	−80.8808	0	0	0	0	0	0	0	0	0	0	0	0	0	0
7	28	24.7638	−80.7331	0	0	0	0	0	0	0	0	0	0	0	0	0	0
8	8.2	24.7711	−80.7936	+++	+	0	+	0	0	0	0	++	0	0	0	x	x
9	8.2	24.7469	−80.7945	+	0	0	0	+	+	+	0	+	+	+	0	x	x
10	37.5	24.6612	−80.9627	0	0	+	0	0	0	0	0	0	0	0	0	x	0
11	26.2	24.6711	−80.9531	0	0	0	0	0	0	0	0	0	0	0	0	0	0
12	10.7	24.7068	−80.9149	0	0	0	0	0	0	0	0	+	0	0	0	x	0
13	3.4	24.7820	−80.8800	0	0	0	0	0	0	+	0	0	0	0	0	x	0
14	35.4	24.7589	−80.7314	0	0	0	0	0	0	0	0	0	0	0	0	0	0
15	29.9	24.7569	−80.7452	0	0	0	0	0	0	0	0	0	0	0	0	0	0
16	6.1	24.7845	−80.7903	+++	+	0	0	0	0	+	0	0	0	0	0	x	x
17	6.1	24.8161	−80.7353	0	0	0	0	0	0	++	0	0	0	0	0	x	0
18	34.7	24.7185	−80.8188	0	0	0	0	0	0	0	0	0	0	0	0	0	0
19	7.3	24.7336	−80.8266	0	0	0	0	0	0	0	0	+++	0	0	0	x	0
20	32.6	24.7311	−80.7891	0	0	0	0	0	0	0	0	0	0	0	0	0	0
21	12.8	24.7417	−80.7822	0	0	0	0	0	0	+	0	+++	+++	0	+	x	x
22	7.6	24.7346	−80.8409	0	0	0	0	0	0	0	0	0	0	0	0	0	0
23	3.7	24.7979	−80.8693	+++	+++	0	0	0	0	0	0	0	0	0	0	x	x
24	2.4	24.8442	−80.8638	0	0	0	0	0	0	0	0	0	0	0	0	0	0
25	35.1	24.7788	−80.6996	0	0	0	0	0	0	0	0	+++	0	0	0	x	0
26	15.2	24.7901	−80.6958	0	0	+	++	0	0	+	++	0	0	0	+	x	x
27	35.1	24.6976	−80.8693	0	0	0	0	0	0	0	0	++	0	0	0	x	0

**Note:**

Data across the sampled sites along the central Florida Keys reported separately for live and dead specimens. Ordinal ranks are as follows: present (+): less than three specimens per m^2^; common (++): three to 10 specimens per m^2^; and abundant (+++): 10 or more specimens per m^2^. The column “total” indicates at which localities live and/or dead specimens were found; x = present, 0 = absent. The column ‘site’ provides site numbers that correspond to site numbers shown on Fig. 1.

Sites were selected to cover common types of habitats occurring along an onshore-offshore gradient with the surveyed sites ranging in water depth from 2.5 to 37.5 m. During each dive, the seafloor was surveyed visually for the presence of live echinoids, echinoid tests (denuded skeletons), test fragments (denuded and partially disintegrated skeletons), and trails produced by shallow-burrowing species. The sampling time for each dive was 15 ± 3 min. The abundance of each species was semi-quantitatively recorded using three ordinal ranks: present (less than three specimens per m^2^), common (three to 10 specimens per m^2^), and abundant (10 or more specimens per m^2^). In addition, divers raked sediment with their hands to a depth of ~15 cm to search for echinoids. The raking was done throughout the duration of the dive whenever soft sediment that could potentially host echinoids was present. At each site, exemplar specimens were collected for each encountered species. Upon surfacing, specimens were stored on ice and transferred into 70% ethanol for soft tissue fixation. After multiple days of storage in ethanol, the specimens were air dried. Length measurements were collected for complete specimens along the longitudinal (anterior-posterior) axis. Specimens are stored in the Division of Invertebrate Zoology at the Florida Museum (University of Florida, Gainesville, Florida) under consecutive repository numbers from UF-Echinodermata-24037 to UF-Echinodermata-24069. The final dataset summarizing survey is provided in Appendix 1.

It should be noted here that whereas all species reported in this study represent intermediate-bodied to large-bodied species (>3 cm in test length), our experience in conducting similar surveys elsewhere (*e.g*., Nebelsick & Kowalewski, 1999; Grun, Sievers & Nebelsick, 2014) indicate that small echinoids can be detected on the sediment surface in SCUBA surveys, including specimens as small as 1 mm in length. However, small-bodied live specimens are easier to miss, and dead tests are more likely to disintegrate during hand-raking. Thus, while small-bodied species are detectable, they may be underrepresented or even missed (especially dead specimens) in SCUBA surveys.

The tests of surveyed echinoids are expected to vary in their intrinsic durability across species. This variability in test integrity can potentially impact their post-mortem survival and the resulting abundance in dead assemblages. To assess the potential durability of tests, each species was assessed in terms of relative test thickness and structural reinforcements provided by internal support structures. Test thickness was scored from 0 (thin) to 3 (thick) and structural reinforcements were scored from 0 (support structures absent) to 3 (support structures well developed). The overall durability score was estimated as an arithmetic mean of the thickness and reinforcement scores (Appendix 2).

Occurrences of echinoids in the region, documented in surveys conducted prior to this study (Appendix 3) were downloaded (10/25/2021) from the iDigBio database aggregator (https://www.idigbio.org/portal/search). Longitude and latitude ranges (W 82.0–80.3 and N 24.4–25.2) were defined by the regional study area map (Fig. 1; Appendix 4) and used to restrict the geographic scope of the search. The word ‘echinoidea’ was used as a keyword. Subsequently, the downloaded data were vetted to limit data to irregular echinoid species and species names were reassessed (10/25/2021) using WORMS (https://www.marinespecies.org). Fossil occurrences were excluded. The results were cross-checked against genus-targeted searches (*e.g*., ‘*Meoma’* instead of ‘echinoidea’) and the outputs were generally consistent.

Maps were generated using custom-written R scripts with shoreline coordinates downloaded from https://gnome.orr.noaa.gov/goods/tools/GSHHS/coast_subset on 09/05/2021. Ordinal rank abundances for live and dead specimens were computed for each species by summing up semi-quantitative ordinal scores defined above. The resulting estimate provided a semi-quantitative ordinal proxy that combined frequency of occurrences and semi-quantitative ordinal rank abundance information. Results were also evaluated by downgrading ordinal rank abundance down to simple occurrence (presence-absence) data. That is, the presence-absence data derived from ordinal scores were used to assess if analytical outcomes were sensitive to analytical resolution at which the data were examined. A statistical agreement between ordinal rank abundance of live and dead specimens was evaluated using the Spearman Rank Correlation test. The Spearman Rank Correlation is a standard measure of live-dead concordance used in fidelity studies (*e.g*., Kidwell, 2007) and is particularly appropriate here given the ordinal rank variables used to measure echinoid abundance. Alternatively, Kendall Rank Correlation could be used. However, because past fidelity studies used the Spearman measure of correlation, Kendall Rank Correlation was deemed less useful in terms of comparability to the previous literature. Differences in distribution/frequency of echinoids between shallow and deep habitats were assessed using the Chi-Square Heterogeneity test. Because some of the chi-square analyses were based on tables with low expected frequencies, a Monte Carlo test was employed (Hope, 1968), as implemented in “chisq.test” function in R (R Core Team, 2021). The significance level of α = 0.05 was used to make statistical decisions. All plots, numerical analyses, and statistical tests were performed using custom written scripts in R (R Core Team, 2021, Appendix 5).

The Live-dead fidelity analysis aims to assess the agreement between live communities and sympatric dead assemblages, including concordance in faunal composition (*e.g*., Kidwell, 2007), sample-level diversity/evenness (*e.g*., Olszewski & Kidwell, 2007), and spatial trends (*e.g*., Tyler & Kowalewski, 2017).

## Results

### Assemblage-level patterns

**Figure 2:**
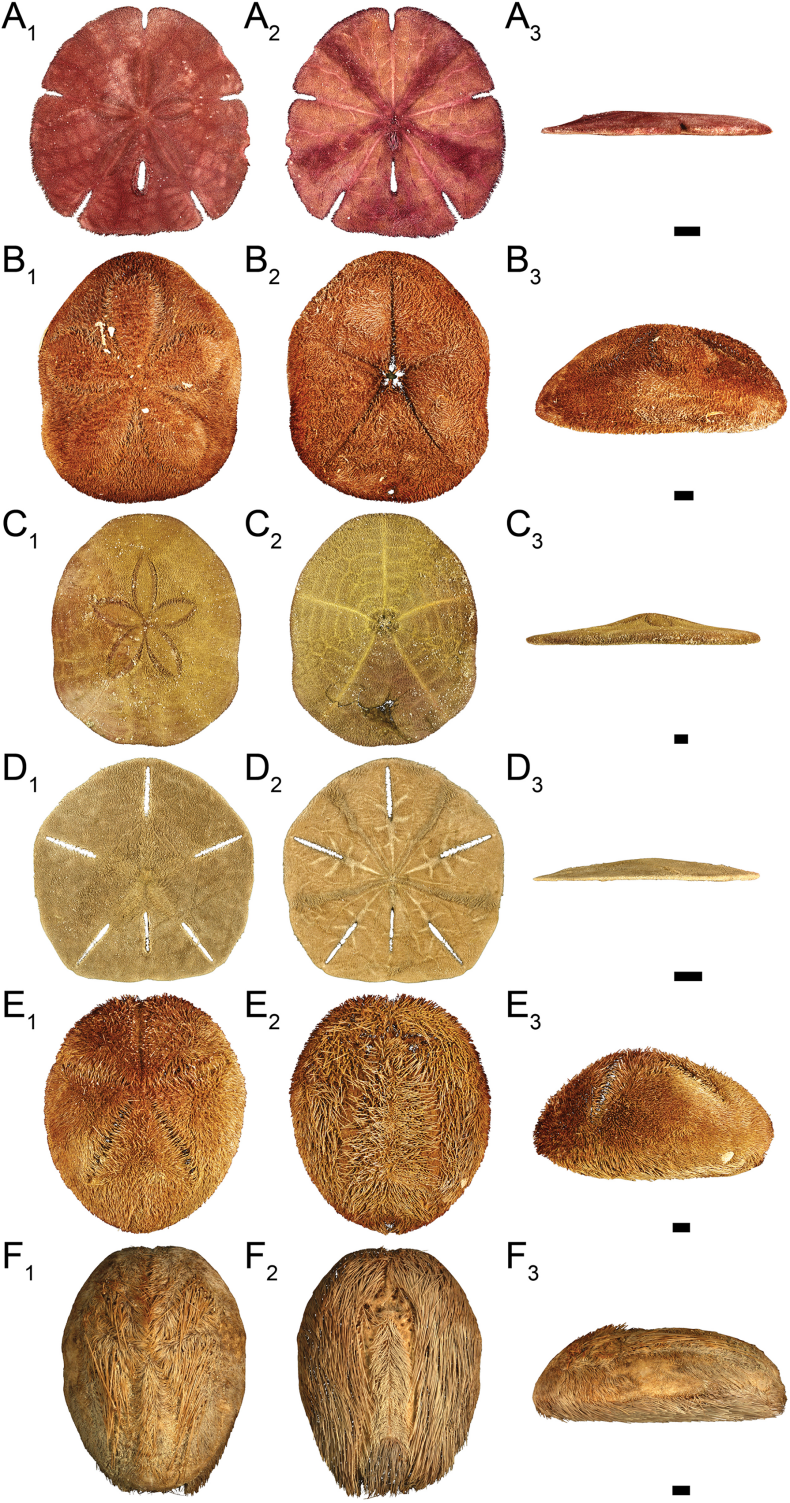
Clypeasteroid and spatangoid echinoids observed in the study area. (A) *Encope michelini* in (A1) aboral view, (A2) oral view, and (A3) lateral view. (B) *Clypeaster rosaceus* in (B1) aboral view, (B2) oral view, and (B3) lateral view. (C) *Clypeaster subdepressus* in (C1) aboral view, (C2) oral view, and (C3) lateral view. (D) *Leodia sexiesperforata* in (D1) aboral view, (D2) oral view, and (D3) lateral view. (E) *Meoma ventricosa* in (E1) aboral view, (E2) oral view, and (E3) lateral view. (F) *Plagiobrissus grandis* in (F1) aboral view, (F2) oral view, and (F3) lateral view. Scale bar = 1 cm.

**Figure 3:**
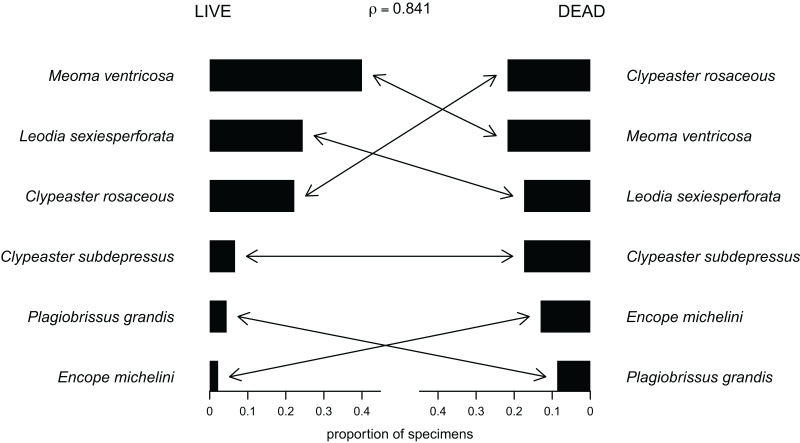
Compositional fidelity of live and dead echinoid assemblages with data pooled across all sites and sampling events. Species ubiquity was measured by summing up ordinal scores across all sites and then converting summed scores into proportions. The Spearman rank correlation rho is reported above the chart.

Out of the 27 surveyed sites, irregular echinoids were encountered at 17 sites (63% of sites) and a total of six species were identified (Fig. 2; Table 1). These included four clypeasteroid species (*Clypeaster rosaceus*, *Clypeaster subdepressus*, *Encope michelini*, and *Leodia sexiesperforata*), and two spatangoid species (*Meoma ventricosa* and *Plagiobrissus grandis*). At all sites, at which irregular echinoids were present, one species was dominant, and in some cases only one species was observed (Table 1). However, at five sites three or more species co-occurred (sites 5, 8, 9, 21, 26) and in one case a total of five species were observed within a single patch of sand (site 9). Out of 17 sites in which echinoids were observed, seven sites were characterized by abundant presence of echinoids, with visually estimated densities exceeding 10 specimens per m^2^. Dead echinoid tests and test fragments were found at nine sites (33% of sites) and abundant tests were found at two sites (Table 1). Dead remains were found at sites where live echinoids were observed. The sturdiness of echinoid tests did not seem to an overriding factor in controlling the abundance of dead tests. In places where alive echinoids with a sturdy test were abundant, bare tests were typically found in higher numbers as well (Table 1). This pattern is similar for echinoids with a low sturdiness rank. However, durability may have played some role in preservation because the most robust taxon (*Clypeaster rosaceus*) ranked higher in dead assemblages than in live assemblages, whereas the much more fragile taxon (*Meoma ventricosa*) was ranked lower in dead assemblages comparing with live assemblages (Fig. 3).

Dense live populations were invariably dominated by one species, including *Meoma ventricosa* at site 21, *Leodia sexiesperforata* at site 5, and *Clypeaster rosaceus* at site 8 and site 18.

**Figure 4:**
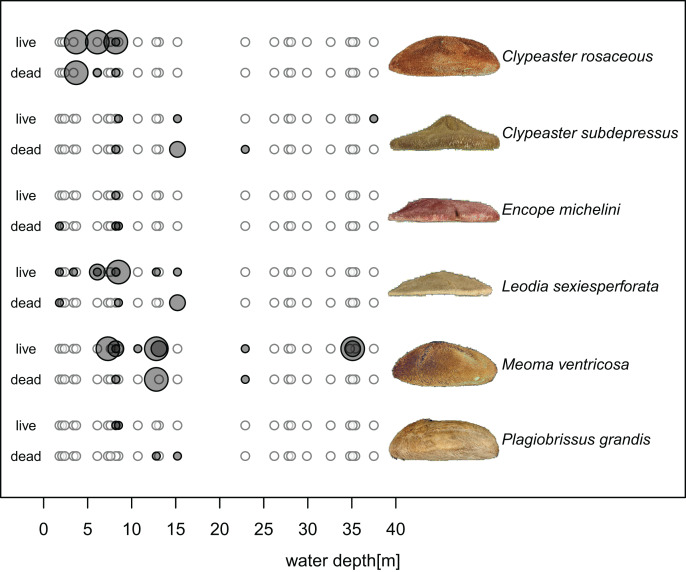
Bathymetric distributions of live and dead echinoids across sites along an onshore-offshore bathymetric gradient. Open symbols indicate sites at which a given species is absent and gray symbols indicate sites at which a given species is present. Symbol size scaled based on ordinal ranking of population abundance: small symbols = rare; intermediate symbols = common; large symbols = abundant.

Echinoids were more widespread, abundant, and diverse in shallower waters (<20 m), including seagrass and algae meadows, open sand flats, and backreef settings, but less common in deeper forereef habitats. Specifically, out of 16 sites sampled in shallow subtidal, Hawk Channel, and backreef habitats, echinoids were present at 13 sites (81%) and abundant at six sites (38%). Multiple species were observed at six sites (38%). In contrast, in deeper forereef habitats (>20 m), echinoids were observed at only four out of 11 sites (36%) and restricted to rare monospecific occurrences of *Meoma ventricosa* and *Clypeaster subdepressus*, with only one site (9%) characterized by abundant presence of *M*. *ventricosa*. The observed differences between shallow habitats and forereef habitats (Fig. 4) were statistically significant in terms of the proportion of sites at which echinoids were present (Chi-Square Test, Chi-Square = 5.7, *p* = 0.039, based on 100,000 replicate Monte Carlo samples).

In addition to echinoids themselves, symbiotic pea crabs (Pinnotheridae) were observed on multiple specimens. They were particularly common on larger species such as *Meoma ventricosa*. Some of the dead echinoid tests included singular circular holes likely recording drilling predation by cassid gastropods. Parasitic eulimid gastropods, that are known to be associated with echinoid hosts, were not observed.

### Species-level patterns

**Figure 5:**
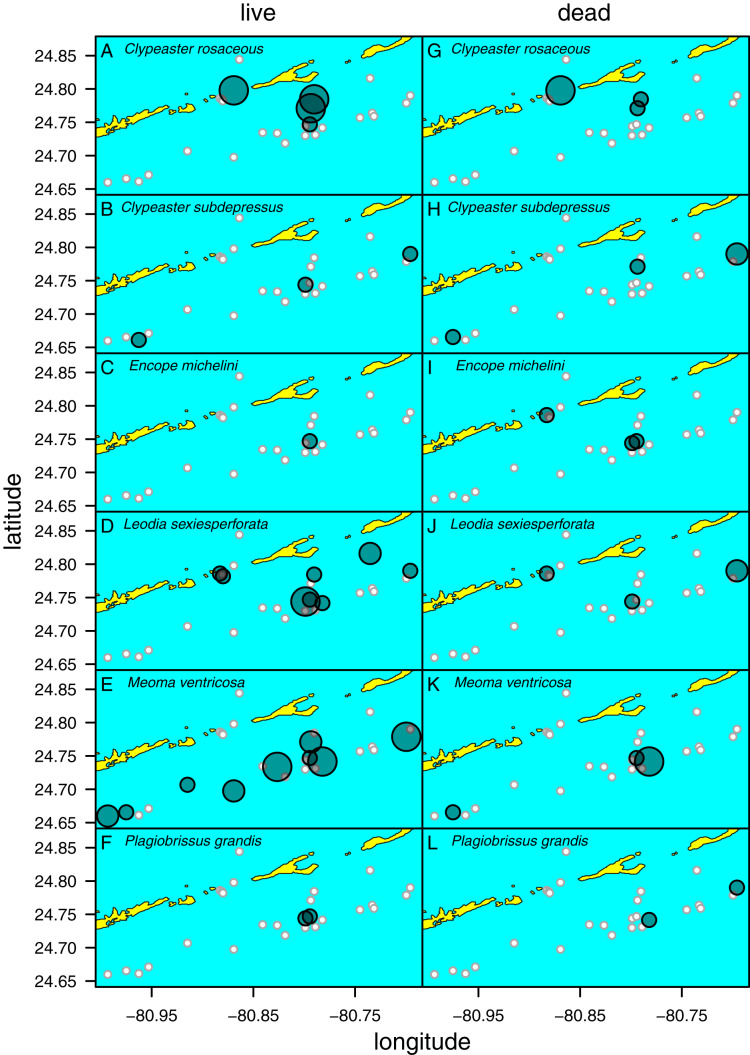
Distribution of six species of irregular echinoids observed in the study area. Each row of two panels represents one species, with the left panel depicting distribution of live echinoids and the right panel depicting presence of dead echinoid remains. Symbol size scaled based on ordinal ranking of population abundance: small white circles = absent; small gray symbols = rare; intermediate gray symbols = common; large gray symbols = abundant.

*Clypeaster rosaceus* (Fig. 2A) was found alive at four sites (Fig. 5A; Table 1) that were characterized by sparse seagrass and algae meadows. All four sites were shallow (<10 m water depth). At two sites, dense populations (>10 specimens per m^2^) were observed. Dead tests co-occurred at three out of the four sites, but only at one site dead tests were abundant. Collected specimens ranged in test length from 39.5 to 137.8 mm with median length of 119.1 mm (*N* = 89). All specimens observed alive were epifaunal with their oral side located around the sediment-water interface. The aboral side of *C. rosaceus* was covered with dead seagrass, debris, and shells. Trails produced by actively moving *C. rosaceus* were observed directly behind active animals with a trail length of up to about two test length. Older trails were rarely discernable.

*Clypeaster subdepressus* (Fig. 2B) was found alive at three sites that represented open sand flats (Fig. 5B; Table 1). These three sites ranged from shallow (<10 m depth) to deeper (~38 m depth) habitats. At all sites, the species was uncommon (<three specimens per m^2^). Dead tests co-occurred with live individuals at one of the three sites. Dead tests were observed at two additional sites at which no live specimens were observed. The measured specimen was 117.5 mm in test length. All specimens observed alive were shallow infaunal to semi-infaunal burrowers with the echinoid body usually penetrating no more than the uppermost 5 cm of sediment. An aboral part of the test was either exposed above the surface or barely covered by a very thin blanket of surficial sediment.

*Encope michelini* (Fig. 2C) was found alive at one site that represented open sand flats (Fig. 5C; Table 1). This site was shallow (8.2 m) with only one live specimen found. Dead tests co-occurred with the live individual at this site. Dead tests were observed at two additional sites at which no live specimens were observed. The live specimen length was 114.1 mm. The observed live specimen was a shallow infaunal burrower with its body being only covered by a thin sediment layer and its outline well visible through the sediment. Visible trails were observed behind the actively moving individual.

*Leodia sexiesperforata* (Fig. 2D) was the most widespread species among recorded clypeasteroid echinoids in the surveyed area. This species was found alive at eight sites that represented open sand flats (Fig. 5D; Table 1). The eight sites ranged from coastal (<2 m depth) to shallow subtidal (~15 m) habitats. At one site, the population was dense (>10 specimens per m^2^), another site was characterized by intermediate population density (three to 10 specimens per m^2^). At six sites, populations were sparse (<three specimens per m^2^). Dead tests co-occurred with live individuals at three sites. Collected specimens ranged in test length from 29.7 to 103.3 mm, with median length of 75.6 mm (*N* = 57). All specimens observed alive were found burrowed in the sediment down to 10–15 cm. Occasionally, individuals of this species were found just below the sediment-water interface.

*Meoma ventricosa* (Fig. 2E) was by far the most common spatangoid species in the surveyed area. This species was found at nine sites that represented sandy habitats (Fig. 5D; Table 1). The nine sites ranged from shallow (<10 m depth) to deeper (~35 m depth) habitats and included three sites where the species was abundant, three sites where the species was common, and three sites where the species was rare. Dead tests co-occurred with live individuals at three sites. Collected specimens ranged in test length from 115.8 to 142.2 mm, with median length of 126.2 mm (*N* = 31). All specimens observed alive were usually found buried with the apical system covered by a thin layer (less than 1 cm thick) of sediment, especially at the shallow sites (<30 m depth). At deeper sites, *Meoma ventricosa* was found only partly buried or even moving on the sediment surface (an unusual daytime behavior for this species). This is in contrast to shallower sites, at which *Meoma ventricosa* was observed buried with the apical system sometimes exposed to the water column. In those settings, this spatangoid produced well-recognizable furrows in the sediment due to its burrowing behavior.

*Plagiobrissus grandis* (Fig. 2F) was the rarest spatangoid in the surveyed area. This species was found alive at two sites that represented sandy flats (Fig. 5F; Table 1). The two sites were shallow to intermediate (eleven and 15 m depth). At both sites, the specimens were observed as single occurrences. Dead tests did not co-occur with live individuals, but two additional sites revealed single dead specimens. The measured specimen was 140.4 mm in test length. All specimens observed alive were infaunal and lived deeply buried between 10 and 20 cm sediment depth. Due to its deep burrowing behavior, the authors assume that this species is underrepresented in this study. Trails were not observed in direct vicinity of live specimens.

### Live-dead patterns

For data pooled across all sites, there is a complete compositional agreement between species found dead and alive: the same six species were observed. In addition, the three species that are most common in the life assemblages are also the most common in the death assemblage (Fig. 3). When using semi-quantitative estimates of echinoid abundance, the compositional fidelity measured as Spearman Rank Correlation is high and statistically significant (*rho* = 0.84, *p* = 0.036). When estimates are reduced to presence-absence data, the correlation is still positive, but weak and statistically insignificant (*rho* = 0.39, *p* = 0.44).

**Figure 6:**
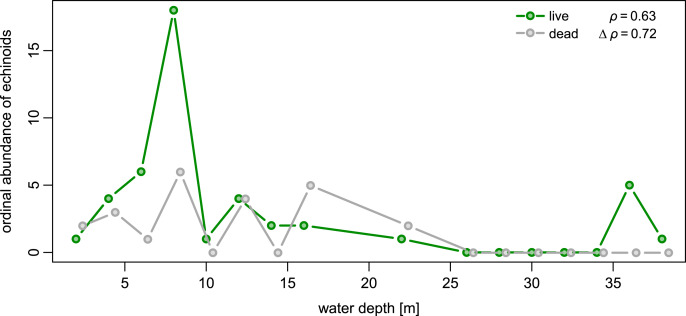
Bathymetric distribution of live and dead echinoids with sites binned into 2 m depth intervals. Both live and dead echinoids occurred more frequently and were more abundant at shallow sites and less common at deeper sites. Spearman rank correlations reported in the top right corner of the plots are significant for both the raw and detrended data (*i.e*., rank correlation of the first differences of the two compared spatial series).

Spatial fidelity was high at the assemblage level. Live and dead echinoids co-occurred at nine sites and were both absent at 10 sites. Live echinoids were present while dead tests were absent only at eight out of 27 sites. However, when dead specimens were observed (nine sites), live specimens were always found (Fig. 5; Table 1). Dead and live echinoids representing the same species co-occurred at eight out of nine sites at which both dead and live specimens were present. The bathymetric distribution was also congruent. Both live and dead echinoids were more frequent and more abundant in shallow habitats when comparing to the more offshore forereef settings (Fig. 4). There was a high and significant Spearman Rank Correlation between ordinal abundance of live and dead echinoids along the sample bathymetric gradient (Fig. 6). The correlation observed for raw data (*rho* = 0.63, *p* = 0.009) increased after detrending the two compared spatial series (*rho* = 0.72, *p* = 0.002) further supporting high spatial/bathymetric live-dead fidelity at the assemblage level. Spatial and bathymetric fidelity was also high at species level (Figs. 4 and 5), although dead remains were occasionally found at sites for which live specimens of the same species were not observed and *vice versa* (Figs. 4 and 5).

**Figure 7:**
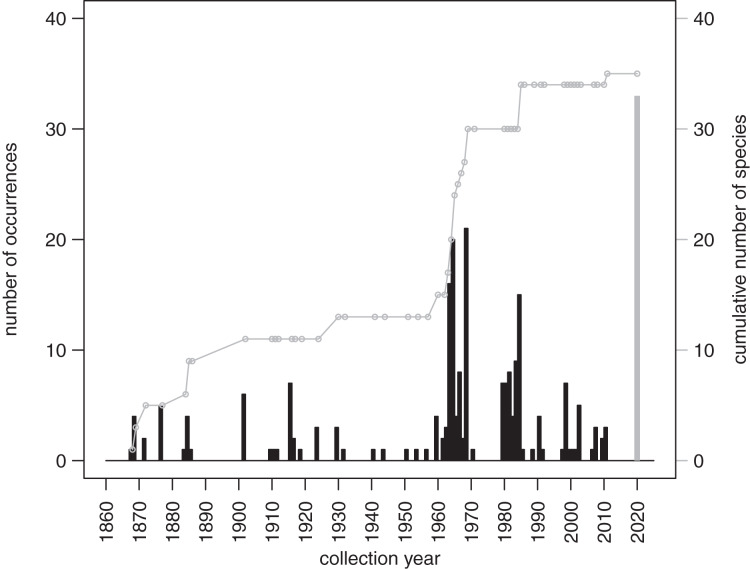
Temporal distribution of sampling events around Florida Keys as archived in online databases aggregated by iDigBio. Black bars represent the number of occurrences of irregular echinoids per year. A single gray bar represents the number of occurrences reported in this study. A gray line represents cumulative growth in the total number of species of irregular echinoids documented in the region. As of today, a total of 37 species of irregular echinoids were reported from the region (see Fig. 8 for the list of species and their rank occurrence).

The records from prior research indicate that 257 occurrences of irregular echinoids identified to genus or species level are currently archived in databases (Appendix 3), with uneven geographic and bathymetric coverage across the study region (Fig. 1). Distribution of sampling events over the last decades (Fig. 7) indicates that most of the occurrences resulted from collecting events in the 1960s and 1980s. The first cluster of sampling events in the 1960s reflects extensive sampling efforts by Kier & Grant (1965) and Chesher (1969). The second cluster dates back to 1984 and 1985, a time interval that directly follows the die off of *Diadema antillarum* (Lessios et al., 1984; Lessios, Robertson & Cubit, 1984). This massive mortality event, which likely started in January 1983 (Lessios, 2016), may have triggered a spike in echinoid surveys. There is a general decline in occurrence reports toward the present.

**Figure 8:**
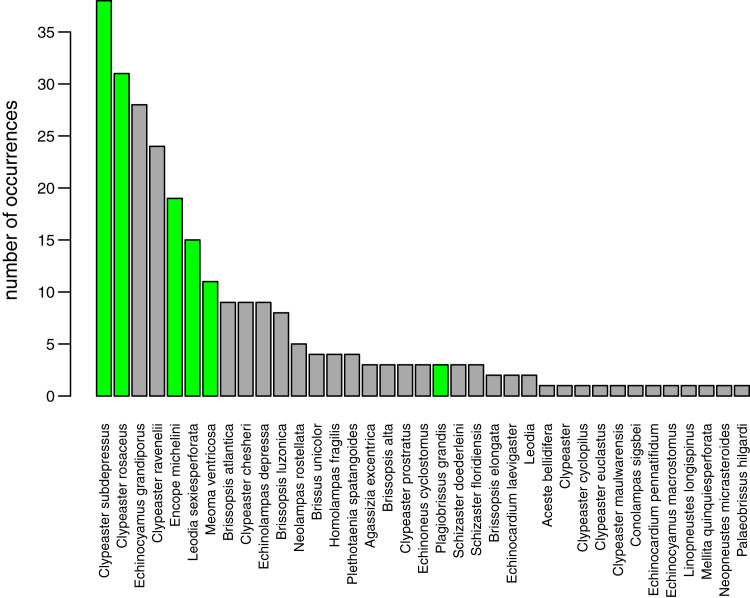
Irregular echinoids documented around Florida Keys in the past studies and surveys (see Fig. 1 for geographic distribution of sites). The species are plotted in rank order of the number of reported occurrences. Green bars indicate species that were found in the current survey.

A total of 37 species of irregular echinoids were reported in the study area (Fig. 8) with the last species newly encountered in the region added in 2011 (Fig. 7). The 10 species that were most common in terms of occurrences were *Clypeaster subdepressus* (38 occurrences), *Clypeaster rosaceus* (31), *Echinocyamus grandiporus* (28), *Clypeaster ravenelii* (24), *Encope michelini* (19), *Leodia sexiesperforata* (15), *Meoma ventricosa* (11), *Brissopsis atlantica* (nine), *Clypeaster chesheri* (nine), and *Echinolampas depressa* (nine). Except for *Plagiobrissus grandis*, the species reported in our survey all represent taxa that were among the top 10 in the region (Fig. 8).

## Discussion

### Neontological implications

The new survey of live and dead echinoids points to a widespread presence of multiple species of clypeasteroid and spatangoid echinoids along the central part of Florida Keys. All six species reported here were previously documented in the region and five of those taxa were among the seven species most frequently encountered in the past (Fig. 8). Moreover, the present-day faunal assemblages resemble closely faunal assemblages that were reported by Kier & Grant (1965) about 60 years ago from a comparable array of habitats in the Key Largo area, 60 km northeastward of our study area. Multiple surveys conducted during the Key Largo study revealed a total of eight clypeasteroid and spatangoid species, including the same six species that were identified in the current survey. The two additional species reported from Key Largo (*Brissus unicolor* and *Schizaster floridiensis*) were rare or represented by dead tests only. These historical comparisons suggest that the present-day irregular echinoid fauna is comparable in its taxonomic composition to faunal associations documented in surveys and case studies conducted in the regions in the 20^th^ century, predominately in 1960s and 1980s (Fig. 7).

The recent survey suggests that many aspects of echinoid biology and ecology have remained unchanged over the last 60 years. In most cases, echinoid species were observed in spatially constrained patches dominated by single species (*e.g*., Smith, 1981; Highsmith, 1982; Tyler et al., 2018). The survey also documented presence of biotic interactions known to be commonly affecting irregular echinoids, including predation by cassid snails (*e.g*., Nebelsick & Kowalewski, 1999; Grun, Sievers & Nebelsick, 2014; Grun, Kroh & Nebelsick, 2017; Grun et al., 2018; Grun, 2017; Meadows, Fordyce & Baumiller, 2015; Tyler et al., 2018) and infestation by symbiotic pea crabs (*e.g*., George & Boone, 2003). The same types of biotic interactions were documented in the region multiple decades ago (Kier & Grant, 1965; Chesher, 1969). The observed behaviors of individual species, including tiering, mobility, and other characteristics, are in line with the ecological knowledge for the six observed species (*e.g*., Seilacher, 1979), except for an intriguing epifaunal daytime mode of live observed for *Meoma ventricosa* in the more offshore, forereef settings. The bathymetric and habitat distribution patterns are also remarkably consistent with past studies. As in the case of previous studies, most of the species were observed in back reef and coastal habitats (Kier & Grant, 1965), whereas the only irregular echinoid relatively common in the deeper, forereef habitat was *Meoma ventricosa* (Kier & Grant, 1965; Chesher, 1969).

In summary, the similarities between the recent survey and past studies suggest that neither faunal composition of dominant taxa, nor spatial distribution and ecology of the common species appear to have undergone any substantial changes over the last 60 years. This is remarkable given that the study area has been intensely affect by local human stressors and global environmental changes. Multiple major hurricanes such as Andrew 1992, Katrina 2005, Wilma 2005 (Malmstadt, Scheitlin & Elsner, 2009) disturbed coastal habitats recently and multiple local stressors related to heavy urbanization, tourism, and local fishery have been continuously impacting the region over the last several decades (*e.g*., Lutz, 2006; Smith, Purcell & Barimo, 2007). However, this study suggests that local populations of irregular echinoids (and thus ecosystem services that they provide) may have remained largely unchanged despite multiple decades of local and global environmental impacts. It should be stressed here that those results should not be used to dismiss the negative impact of human-induced environmental changes in Florida Keys. There are numerous well documented studies demonstrating that the local ecosystems have declined dramatically in recent decades due to direct and indirect human activities, including reefs, wetlands, water quality, and aquatic or water-associated life (*e.g*., Fourqurean & Zieman, 2002; Smith, Purcell & Barimo, 2007). However, the results of this survey suggest that the documented irregular echinoid species may be more resilient to environmental impacts than other organisms. Consequently, they deserve attention as resilient benthic macro-organisms and important ecosystem engineers that keep thriving across a wide range of the Florida Keys habitats and keep contributing in terms of ecosystem services that benefit local benthic ecosystems.

### Paleontological implications

The high fidelity indicated by live-dead comparisons suggests that dead remains of echinoid may archive spatial distribution and taxonomic composition of local echinoid populations. However, this interpretation needs to be treated cautiously because it is based on one study area. Also, multiple causative explanations can be provided to explain the observed congruence. Specifically, two alternative hypotheses can be postulated to explain the high congruence of living communities and death assemblages. Hypothesis 1: if echinoid tests represent a time-averaged assemblage that accumulated over multiple decades or centuries, the high congruence could indicate that local populations of echinoids have been remarkably stable in terms of their spatial distribution and taxonomic composition. The striking similarity with the surveys of Kier & Grant (1965) conducted about 60 years ago are consistent with hypothesis 1, as are observations from other regions documenting monospecific populations of echinoids persisting in small patches for multiple decades (*e.g*., Smith, 1981; Highsmith, 1982; Tyler et al., 2018). Previous surveys (Appendix 3) of sites close to our survey sites (Fig. 1) uncovered a comparable suite of irregular echinoid, including *Clypeaster rosaceus*, *Leodia sexiesperforata*, and *Encope michelini*. However, previous surveys also reported *Echinocardium* (Appendix 3), which has not been noted in our surveys.

The alternative hypothesis 2 is based on taphonomic filters and states that echinoid remains all came from individuals that died in recent months or years. There is growing evidence that irregular echinoid tests perish quickly and cannot form extensively time-averaged archives of local echinoid populations. More recently, direct evidence supporting taphonomic fragility of skeletal remains of irregular echinoid were provided *via* radiocarbon dating of individual tests of *Leodia sexiesperforata* on a shallow carbonate platform in Bahamas (Kowalewski et al., 2018). The dating effort indicated that all sampled tests came from individuals that died in the few previous years, even though the sympatric mollusk shells collected from the same sediments were time-averaged over multi-millennial time scales (Kowalewski et al., 2018). Under the hypothesis 2, echinoid tests do not survive substantial lateral transport or persist for decades or centuries around the sediment surface, and consequently, the high spatial and compositional fidelity reflects the fact that the great majority of dead remains found during the surveys represent recently deceased specimens that lived in the area. This hypothesis is also consistent with taphonomic studies on regular echinoids (*e.g*., Kidwell & Baumiller, 1990; Greenstein, 1991).

However, Nawrot et al. (2022) documented recently extensive time-averaging for small clypeasteroid echinoids. Thus, the hypothesis 2, even if proven correct for the study area, should be extrapolated with caution to other echinoid taxa and other environmental settings.

More generally, the data available currently cannot resolve which of those two hypotheses is more likely to be correct. This is because the observed results could be produced if echinoid patches persist through time and/or dead remains disappear quickly. However, regardless of its causative explanation, the observed spatial and compositional fidelity has two potential implications. First, the incipient fossil record currently forming around sediment surface may provide a faithful representation of living echinoid populations, both in terms of spatial distribution and faunal composition. Thus, if the surficial sediment were to be preserved in a rapid burial event, the resulting fossil record would archive echinoid populations with high spatial and compositional fidelity. Second, the dead remains track closely living populations and thus their presence provides a circumstantial line of evidence for the presence of living echinoids in the area. In the case of the study area, whenever dead remains were found, living echinoids were always observed. Comparative live-dead studies of irregular echinoids in other climatic and depositional settings across a broader suite of taxa is needed to confirm if these observations are valid as a broader generalization.

Finally, occurrences of echinoid remains may constitute valuable data that can enhance bio-inventorying efforts based on surveys of live individuals.

Whereas the abundance of dead material on species level might be skewed towards more robust skeletons, the results of this study indicate that species that are more abundant alive, are generally also more abundant in death assemblages (Fig. 3).

### Bio-inventorying implications

The survey of the literature and databases indicates that most of the efforts focused on bio-inventorying echinoids around Florida Keys took place in the mid-to-late 20 century and declined dramatically over the most recent decades (Fig. 7). This dearth of recent surveys points to the critical importance of allocating time and resources toward resurveying marine habitats, especially in areas that experience intense and diverse human impacts. The recent surveys such as the one reported here not only allow for comparative assessment with past surveys and archived occurrence records, but also provide an important reference point for future reassessment and monitoring efforts.

## Conclusions

1. Irregular echinoids are present in 63% of surveyed sites in the Florida Keys.
2. When echinoids are present, their abundance is typically high (>10 individuals per m^2^).
3. Six irregular echinoid species were identified: *Clypeaster rosaceus*, *Clypeaster subdepressus*, *Encope michelini*, *Leodia sexiesperforata*, *Meoma ventricosa*, and *Plagiobrissus grandis*.
4. The distribution and faunal composition of echinoids is consistent with observations made 50 years ago (Kier & Grant, 1965) suggesting that despite numerous anthropogenic stressors affecting the region the echinoid fauna has remained relatively unchanged over the recent decades.
5. Up to five species co-occurred within single sites in shallow waters (<20 m), but typically only one species was dominant.
6. Deeper sites (>20 m) appear to be restricted to rare monospecific occurrences of *Meoma ventricosa* and *Clypeaster subdepressus*.
7. Dead echinoid tests typically co-occur with live specimens but are less frequent than live specimens.

## Supplemental Information

10.7717/peerj.14245/supp-1 Supplemental Information 1 Dataset resulting from surveys reported in this study. Ordinal rank codes are numerical codes X.Y, where X is the ordinal rank for live specimens and Y is the ordinal rank for dead specimens. For example, a value of 2.1 for a given taxon means that the live specimens of that taxon were common (rank = 2) and dead remains were present (rank = 1).

10.7717/peerj.14245/supp-2 Supplemental Information 2 Sturdiness assignment for echinoid tests. 0 = absent, 1 = low, 2 = intermediate, 3 = high.

10.7717/peerj.14245/supp-3 Supplemental Information 3 iDigBio dataset. Downloaded (10/25/2021) from https://www.idigbio.org/portal/search.

10.7717/peerj.14245/supp-4 Supplemental Information 4 Coastline coordinates downloaded from used to plot maps. Coordinates were downloaded from https://gnome.orr.noaa.gov/goods/tools/GSHHS/coast_subset on September 5 2021. The map boundaries were defined by following decimal coordinates: −82.0W, −80.3W, 24.4N, 25.2N.

10.7717/peerj.14245/supp-5 Supplemental Information 5 R-Code. Custom-written code used for analysis and graphs.

## Additional Information and Declarations

### Field Study Permissions

The following information was supplied relating to field study approvals (*i.e*., approving body and any reference numbers): Permit was issued by the Florida Fish and Wildlife Conservation Commission (SAL 19-2195-SR SAL 18-1294A-SR).

### Data Availability

The following information was supplied regarding data availability: The data and codes are available in the Supplemental Files.
