# Supplementary material for: Spatial distribution, diversity, and taphonomy of clypeasteroid and spatangoid echinoids of the central Florida Keys
Source: PeerJ. 2022 Oct 31;10:e14245. doi: 10.7717/peerj.14245 (PMC9632462; doi:10.7717/peerj.14245)
Supplement: Supplemental Information 5 — Custom-written code used for analysis and graphs. [file peerj-10-14245-s005.docx]

#--------------------------------------------------------------

# Appendix 5

# R script with figures and analysis associated with

# Grun and Kowalewski (2022) Spatial distribution, diversity, and

# taphonomy of clypeasteroid and spatangoid echinoids of the

# central Florida Keys. Revised Manuscript, PeerJ.

#

# Written by M. Kowalewski (U. Florida)

# Last updated: August 16, 2022

#

# NOTE: This script will reproduce all figures and analyses

# except for images inserted on some plots which require

# echinoid images not provided here.

#

# Contact: kowalewski@ufl.edu

#--------------------------------------------------------------

#---------------------------------------------------------------

#----------------- packages, source files, and options --------

#---------------------------------------------------------------

# install.packages('jpeg') #not run

library(jpeg)

# PDF definition

outPDF <- T # set to 'T' or TRUE to output pdf files with figures

pdf.options(paper="special", onefile=TRUE, family='Helvetica',

pointsize=10, encoding="ISOLatin1.enc")

#---------------------------------------------------------------

# LDPlot Function from an unpublished R package "Paleofidelity'

# by M. Kowalewski

LDPlot <- function(live, dead, tax.names, toplimit = 10, barwidth = 150 / toplimit,

col1 = 'black', col2 = 'gray', arr.col = 'black', arr.lty=1,

arr.lwd = 1, cex.axis = 0.7, tck = -0.02, cex.lab = 0.8,

cex.names = 0.95, cex.label = 1, cex.stat = 0.9, font.names = 3,

cor.measure = 'spearman', report=F) {

if (!is.vector(live)) stop('object "live" must be a vector')

if (!is.vector(dead)) stop('object "dead" must be a vector')

if (!is.numeric(live)) stop('object "live" must be numeric')

if (!is.numeric(dead)) stop('object "dead" must be numeric')

if (length(live) != length(dead))

stop('objects "live" and "dead" are of different length')

if (length(live) != length(tax.names))

stop('object "tax.names" differs in length from objects "live" and "dead"')

if (sum((live + dead) > 0) < toplimit)

stop(paste('toplimit = ', toplimit, 'exceeds the total number of non-zero

taxa/variables =', sum((live + dead) > 0), 'set toplimit <=',

sum((live + dead) > 0)))

if (max(table(rank(live)))/length(live) > 0.5)

warning('more than 50% of "live" species/units have the same rank:

all species with tied ranks will be ordered arbirtarily when plotted')

if (max(table(rank(dead)))/length(dead) > 0.5)

warning('more than 50% of "dead" species/units have the same rank:

all species with tied ranks will be ordered arbirtarily when plotted')

# info on top n live and dead units

toplive <- (live[order(live, decreasing = T)] / sum(live)) [1:toplimit]

toplivenames <- tax.names[order(live, decreasing=T)][1:toplimit]

topdead <- (dead[order(dead, decreasing = T)] / sum(dead)) [1:toplimit]

topdeadnames <- tax.names[order(dead, decreasing = T)] [1:toplimit]

dead.live <- as.numeric(topdeadnames %in% toplivenames)

live.dead <- as.numeric(toplivenames %in% topdeadnames)

if (length(which(topdead == 0)) > 0) {

topdeadzero <- which(toplivenames %in% topdeadnames[which(topdead == 0)])

live.dead[topdeadzero] <- 0

}

if (length(which(toplive == 0)) > 0) {

toplivezero <- which(topdeadnames %in% toplivenames[which(toplive == 0)])

dead.live[toplivezero] <- 0

}

# compute correlation coefficients

if (cor.measure != 'all') corest <- round(stats::cor(live, dead, method=cor.measure), 3)

if (cor.measure == 'pearson') cor.label <- bquote(italic(r) == .(corest))

if (cor.measure == 'spearman') cor.label <- bquote(italic(rho) == .(corest))

if (cor.measure == 'kendall') cor.label <- bquote(italic(tau) == .(corest))

if (cor.measure == 'all') {

corest1 <- round(stats::cor(live, dead, method='pearson'), 3)

cor.label1 <- bquote(italic(r) == .(corest1))

corest2 <- round(stats::cor(live, dead, method='spearman'), 3)

cor.label2 <- bquote(italic(rho) == .(corest2))

corest3 <- round(stats::cor(live, dead, method='kendall'), 3)

cor.label3 <- bquote(italic(tau) == .(corest3))

}

# customize info for x axis

max.x <- ceiling(5*max(c(toplive, topdead)))/5

xlim2 <- 2 * max.x * 1.25

if (max.x <= 0.5) {

my.x.at <- c(seq(0, max.x, 0.1), seq(xlim2-max.x, xlim2, 0.1))

my.x.lab <- c(seq(0, max.x, 0.1), seq(max.x, 0, -0.1))

}

if (max.x > 0.5) {

my.x.at <- c(seq(0, max.x, 0.2), seq(xlim2-max.x, xlim2, 0.2))

my.x.lab <- c(seq(0, max.x, 0.2), seq(max.x, 0, -0.2))

}

# plot LD chart

graphics::plot(0, 0, type = 'n', ylim = c(toplimit, 0), xlim = c(0, xlim2),

xlab = '', ylab = '', axes = F)

for(i in 1:toplimit) {

if (live.dead[i] == 0) graphics::lines(c(0, toplive[i]), c(i , i),

lwd = barwidth, lend = 3, col = col2)

if (live.dead[i] == 1) graphics::lines(c(0, toplive[i]), c(i, i),

lwd = barwidth, lend = 3, col = col1)

if (dead.live[i] == 0) graphics::lines(c(xlim2 - topdead[i], xlim2), c(i, i),

lwd = barwidth, lend = 3, col = col2)

if (dead.live[i] == 1) graphics::lines(c(xlim2 - topdead[i], xlim2), c(i, i),

lwd = barwidth, lend = 3, col = col1)

}

graphics::axis(2, labels = toplivenames, at = 1:toplimit, lwd = 0, las = 1,

padj = 0.5, hadj = 1, font = font.names, cex.axis = cex.names)

graphics::axis(4, labels = topdeadnames, at = 1:toplimit, lwd = 0, las = 1,

padj = 0.5, hadj = 0, font = font.names, cex.axis = cex.names)

graphics::axis(1, labels = my.x.lab, padj = -1.5, tck = tck, at = my.x.at,

cex.axis = cex.axis)

graphics::lines(c(0.45 * xlim2, 0.55 * xlim2), c(toplimit, toplimit), lwd = 50,

col = 'white', lend = 3, xpd = NA)

graphics::mtext(side = 1, line = 1.5, text='proportion of specimens', cex=cex.lab)

graphics::mtext(side = 3, line = -1, adj = 0, text = 'LIVE', cex = cex.label, xpd = NA)

graphics::mtext(side = 3, line = -1, adj = 1, text = 'DEAD', cex = cex.label, xpd = NA)

if (cor.measure != 'all') graphics::mtext(side=3, line=-1, text=cor.label, cex = cex.stat, xpd = NA)

if (cor.measure == 'all') {

graphics::mtext(side = 3, line = -1.6, text = cor.label1, cex = cex.stat, xpd = NA)

graphics::mtext(side = 3, line = -1, text = cor.label2, cex = cex.stat, xpd = NA)

graphics::mtext(side = 3, line = -0.1, text = cor.label3, cex = cex.stat, xpd = NA)

}

for(i in 1:toplimit) {

if (dead.live[i] == 1) {

k <- which(toplivenames == topdeadnames[i])

if (live.dead[k] == 1) {

graphics::arrows((xlim2 - topdead[i]) - (0.03 * xlim2), i,

(0.03 * xlim2) + toplive[k], k, length = 0.1,

lwd = arr.lwd, code = 3, col = arr.col, lty = arr.lty)

}

}

}

if (report) {

basics <- c('all species' = length(live),

'total species' = sum(live + dead > 0),

'species shared' = sum(live > 0 & dead > 0),

'shared absent' = sum(live + dead == 0),

'missing dead' = sum(live > 0 & dead == 0),

'missing live' = sum(live == 0 & dead > 0))

pct.basics1 <- basics/basics[1]

pct.basics2 <- basics/basics[2]

sum.basics <- cbind(count=basics, proportion.global=pct.basics1,

proportion.local=pct.basics2)

sample.sizes <- c('total n' = sum(live) + sum(dead),

'total live' = sum(live),

'total dead' = sum(dead))

cors <- c(pearson = stats::cor(live, dead, method='pearson'),

spearman = stats::cor(live, dead, method='spearman'),

kendall = stats::cor(live, dead, method='kendall'))

report.out <- list(summary = sum.basics, "sample info" = sample.sizes,

cor.coeff = cors)

return(report.out)

}

}

#---------------------------------------------------------------

#---------------------------------------------------------------

# function based on function suggested by user:gaspar for removing

# black background from an image at

# https://stackoverflow.com/questions/59861122/change-image-pixel-colors-in-r-and-save-image

change_cols = function(replace_black, replace_white, theimg) {

r_b = col2rgb(replace_black) / 255

r_w = col2rgb(replace_white) / 255

theimg[theimg == 1] <- 2

for (i in 1:3) {

theimg[,,i][theimg[,,i] == 0] <- r_b[i]

}

for (i in 1:3) {

theimg[,,i][theimg[,,i] == 2] <- r_w[i]

}

return(theimg)

}

#--------------------------------------------------------------

#----------------- upload and pre-process echinoid images -----

Mv.img<-readJPEG("Meom.jpg")

Em.img<-readJPEG("Emic.jpg")

Les.img<-readJPEG("Les.jpg")

Pg.img <- readJPEG("Pgr.jpg")

Cs.img <- readJPEG("Clys.jpg")

Cr.img <- readJPEG("Clyr.jpg")

Mv.img2<-change_cols("#FFFFFF", "#000000", Mv.img)

Em.img2<-change_cols("#FFFFFF", "#000000", Em.img)

Les.img2<-change_cols("#FFFFFF", "#000000", Les.img)

Pg.img2<-change_cols("#FFFFFF", "#000000", Pg.img)

Cs.img2<-change_cols("#FFFFFF", "#000000", Cs.img)

Cr.img2<-change_cols("#FFFFFF", "#000000", Cr.img)

#--------------------------------------------------------------

#------------------------ COASTLINE MAP ------------------------

# coastal coordinates downloaded from:

# https://gnome.orr.noaa.gov/goods/tools/GSHHS/coast_subset

cst <- read.csv('Appendix 4.csv', header=F)

plgs <- which(is.na(cst[2,]))

#--------------------------------------------------------------

#---- Upload Grun and Kowalewski echinoid data ----------------

edd2 <- read.csv('Appendix 1.csv', stringsAsFactors = T)

edd2$Depth <- round(edd2$Depth_ft * 0.305, 1)

colD <- round(edd2$Depth)

#--------------------------------------------------------------

#---- Upload and preprocess IDIGBIO data ----------------------

new.occs <- sum(rowSums(edd2[,7:12]>0))

idg <- read.csv('Appendix 3.csv', na.strings = c('', '.', NA))

# restrict to class 'echinoidea'

idg <- idg[which(idg$dwc.class == 'echinoidea'),]

# remove fossil specimens

idg <- idg[-which(idg$dwc.basisOfRecord == 'fossilspecimen'),]

# restrict to relevant families only

idgi <- idg[which(idg$dwc.family %in% c('brissidae',

'cassidulidae',

'clypeasteridae',

'echinocyamidae',

'echinolampadidae',

'eurypatagidae',

'mellitidae',

'echinoneidae',

'eurypatagidae',

'loveniidae',

'maretiidae',

'mellitidae',

'neolampadidae',

'palaeotropidae',

'prenasteridae',

'schizasteridae',

'spatangidae')),]

write.csv(table(idgi$gbif.canonicalName), 'FKspecies.csv')

write.csv(idgi, 'Appendix IDGIBIO Irregular Echinoids.csv')

table(idgi$gbif.canonicalName)

sum(table(idgi$gbif.canonicalName))

#--------------------------------------------------------------

#---- FIGURE 8 ------------------------------------------------

if(outPDF) pdf("Past Species.pdf", height=4.5, width=6)

my.col1 <- 'green1'

my.col2 <- 'darkgray'

temp.par <- par(mar=c(9,5,1,1))

sort(table(idgi$gbif.canonicalName), decreasing = T)

barplot(sort(table(idgi$gbif.canonicalName), decreasing = T),

ylab='number of occurrences', las=2, cex.axis=0.8,

cex.names=0.7, col=c(my.col1, my.col1, my.col2,

my.col2, my.col1, my.col1,

my.col1, rep(my.col2, 12),

my.col1, rep(my.col2, 17)))

par(temp.par)

if(outPDF) dev.off()

#--------------------------------------------------------------

#---- FIGURE 7 ------------------------------------------------

out.hist <- NULL

for (i in seq(1860,2021,1)) {

temp.rws <- which(idgi$Year %in% 1860:i)

if (length(temp.rws) > 0 & length(which(idgi$Year==i) > 0)) {

out.hist <- rbind(out.hist,

cbind(i,

length(table(idgi$gbif.canonicalName[temp.rws]))))

}

}

if(outPDF) pdf("Research History.pdf", height=4.5, width=6)

temp.par <- par(mar=c(5,5,1,5))

out.hist <- rbind(out.hist, cbind(2020,max(out.hist[,2])))

hist(idgi$Year, breaks=seq(1860,2025,1), main='',

las=1, axes=F, col='black',

xlab='collection year',

ylab='number of occurrences', ylim=c(0,40))

points(c(2020,2020), c(0,new.occs), type='l', lwd=4,

col='darkgray', lend=3)

axis(1, at=seq(1860,2025,10),

labels=seq(1860,2025,10), las=2)

axis(2, las=1)

axis(4, las=1, col.lab='darkgray', col='darkgray', col.ticks='darkgray')

mtext(side=4, line=2.5, 'cumulative number of species',

col='darkgray')

points(out.hist, type='o', pch=21, cex=0.6, col='darkgray')

box()

par(temp.par)

if(outPDF) dev.off()

#--------------------------------------------------------------

#---- FIGURE 1 ------------------------------------------------

# color scheme

wt.col <- 'white' # water color

sa.bg.cl <- adjustcolor('gray20',.3) # study area background color

our.site.bg <- 'gray20' # our sites (background color)

our.site.out <- 'white' # our sites (outline color)

land.col <- 'gray80' # color of land area

coast.col <- 'gray20' # color of coastline

old.stud <- 'gray20'

# plot a map of the keys and indicate study area and sites

if(outPDF) pdf("MAP OF KEYS.pdf", height=4.5, width=6)

plot.lim <- rbind(c(-82, 24.4), c(-80.3, 25.2))

plot.lim2 <- t(cst[,2:5]) # full map

plot(plot.lim, type='n', xlab='', ylab='', las=1)

polygon(t(cst[,2:5]), col=wt.col, border=NA)

points(c(-81.8, -81.58), c(25.2, 25.2), type='l', lwd=2, lend=3)

text(-81.68, 25.17, '20 km', cex=0.7)

points(c(-81.9, -81.9), c(25.05, 25.18), type='l', lwd=1.5, lend=3)

points(-81.9, 25.18, pch=17, cex=1.5)

text(-81.9, 25.12, 'N', cex=4, col=wt.col)

text(-81.9, 25.12, 'N', cex=1)

for (i in 2:length(plgs)) {

if (i < length(plgs)) {

a1 <- plgs[i]+1

a2 <- plgs[i+1]-1

polygon(t(cst[,a1:a2]), col=land.col, border=coast.col)

}

}

rect(min(edd2$longitude)-0.02, min(edd2$latitude)-0.01,

max(edd2$longitude)+0.02, max(edd2$latitude)+0.01,

col=sa.bg.cl, border='black', lwd=1)

points(edd2$longitude, edd2$latitude, pch=21,

bg=our.site.bg, col=our.site.out, cex=0.9)

mtext(side=2,'latitude', line=3, cex=1.2)

mtext(side=1,'longitude', line=2.5, cex=1.2)

box()

points(idgi$Long, idgi$Lat, pch=8, cex=0.7, col=old.stud)

points(-82, 25.26, pch=8, cex=0.7, col=old.stud, xpd=NA)

text(-81.8, 25.26, 'previous records', cex=0.7, col='black', xpd=NA)

points(-81.4,25.26, pch=21, bg=our.site.bg, col=our.site.out, cex=0.9, xpd=NA)

text(-81.27, 25.26, 'this study', cex=0.7, col='black', xpd=NA)

if(outPDF) dev.off()

#--------------------------------------------------------------

#--------------------------------------------------------------

# Bathymetric distribution of species CHI-SQUARE TEST

liveE <- floor(edd2[,7:12])

deadE <- 10 * (edd2[,7:12] - floor(edd2[,7:12]))

tx.labels <- gsub('.', ' ', colnames(edd2), fixed=T)[7:12]

shallow <- rowSums(liveE)[which(edd2$Depth<20)]

deep <- rowSums(liveE)[which(edd2$Depth>=20)]

chisq.test(rbind(table((rowSums(liveE>0)>0)[which(edd2$Depth<20)]),

table((rowSums(liveE>0)>0)[which(edd2$Depth>=20)])),

simulate.p.value=T, B=100000)

#--------------------------------------------------------------

#------- FIGURE 5 & SPEARMAN TESTS ----------------------------

if(outPDF) pdf("Fidelity.pdf", height=4, width=7)

temp.par <- par(mar=c(3,11,2,11))

LDPlot(colSums(liveE), colSums(deadE), tax.names=tx.labels,

toplimit=6, cor.measure = 'spearman', cex.names=0.9)

par(temp.par)

if(outPDF) dev.off()

# Spearman Test: fidelity pooled ordinal data

cor.test(colSums(liveE), colSums(deadE), method='spearman')

# Spearman Test: fidelity pooled binary data

cor.test(colSums(liveE>0), colSums(deadE>0), method='spearman')

#--------------------------------------------------------------

#------- FIGURE 5 ---------------------------------------------

mybin <- 2

ldepth <- tapply(rowSums(liveE),

round(1/mybin*edd2$Depth)*mybin, sum)

ddepth <- tapply(rowSums(deadE),

round(1/mybin*edd2$Depth)*mybin, sum)

dld <- ldepth[-1] - ldepth[-length(ldepth)]

ddd <- ddepth[-1] - ddepth[-length(ddepth)]

cor.raw <- cor.test(ldepth, ddepth, method='spearman')

cor.fd <- cor.test(dld, ddd, method='spearman')

cor.raw$p.value

cor.fd$p.value

if(outPDF) pdf("DepthFidelity.pdf", height=4, width=7)

plot(as.numeric(names(ldepth)), ldepth,

pch=21, type='b', col='green4',

bg=adjustcolor('green4', .4), lwd=2,

xlab='water depth [m]',

ylab='ordinal abundance of echinoids')

points(as.numeric(names(ddepth)), ddepth,

pch=21, type='b', col='darkgray', lwd=2,

bg=adjustcolor('darkgray', .4))

mtext(side=3, line=-1.5, adj=0.95, cex=0.9,

bquote(rho == .(round(cor.raw$estimate,2))))

mtext(side=3, line=-2.5, adj=0.95, cex=0.9,

bquote(Delta~rho == .(round(cor.fd$estimate,2))))

if(outPDF) dev.off()

#--------------------------------------------------------------

#------- FIGURE 3 ---------------------------------------------

##### Figure live-dead bathymetric distribution across sites

typeE <- liveE # live

typeD <- deadE # dead

site.col.out <- 'gray50'

site.col.bg <- 'white'

site.group <- as.factor(rowSums(typeE+typeD)>0)

typeE2 <- typeE[,order(colnames(typeE))]

typeD2 <- typeD[,order(colnames(typeD))]

op <- par(mar=c(1,0,0,1), oma=c(0,0,0,0))

if(outPDF) pdf("FIGURE_DEPTH_LIVE.pdf", height=5.5, width=6.5)

plot(0,0, xlim=c(-2,70), ylim=c(0.5, 6.5), type='n',

xlab='', axes=F, ylab='')

for (i in 1:6) {

is.E <- which(typeE2[,i] > 0)

is.D <- which(typeD2[,i] > 0)

points(edd2$Depth[-is.E], rep(7-i+0.2, nrow(edd2))[-is.E], cex=1.1, pch=21,

col=site.col.out, bg=adjustcolor(site.col.bg,.5))

points(edd2$Depth[-is.D], rep(7-i-0.2, nrow(edd2))[-is.D], cex=1.1, pch=21,

col=site.col.out, bg=adjustcolor(site.col.bg,.5))

points(edd2$Depth, rep(7-i+0.2, nrow(edd2)), cex=typeE2[,i], pch=21,

col='black', bg=adjustcolor('black', 0.4))

points(edd2$Depth, rep(7-i-0.2, nrow(edd2)), cex=typeD2[,i], pch=21,

col='black', bg=adjustcolor('black', 0.4))

text(52, 7-i, tx.labels[order(colnames(typeE))][i],

font=3, cex=0.9, adj=0, xpd=NA)

text(-2, 7-i+0.2, "live", cex=0.8, xpd=NA)

text(-2, 7-i-0.2, "dead", cex=0.8, xpd=NA)

}

axis(1, at=seq(0,40,5), labels=seq(0,40,5))

mtext('water depth[m]', side=1, line=2.5)

# Now add images with custom coordinates

rasterImage(Cr.img2,39,5.7,52,6.3, xpd=NA)

rasterImage(Cs.img2,39,4.7,52,5.3, xpd=NA)

rasterImage(Em.img2,39,3.7,52,4.3, xpd=NA)

rasterImage(Les.img2,39,2.7,52,3.3, xpd=NA)

rasterImage(Mv.img2,39,1.7,52,2.3, xpd=NA)

rasterImage(Pg.img2,39,0.7,52,1.3, xpd=NA)

box()

par(op)

if(outPDF) dev.off()

#--------------------------------------------------------------

#--------------------------------------------------------------

# Map of all sites (unpublished)

mycol.F <- colorRampPalette(c("gray100", 'gray10'))

mycols <- mycol.F(max(colD)+1)

if(outPDF) pdf("FIGURE_SITES.pdf", height=4.5, width=6)

plot(edd2$longitude, edd2$latitude, xlab='', ylab='',

las=1, cex.axis=0.8, ylim=c(24.65,24.87))

polygon(t(cst[,2:5]), col='white', border=NA)

box()

for (i in 2:length(plgs)) {

if (i < length(plgs)) {

a1 <- plgs[i]+1

a2 <- plgs[i+1]-1

polygon(t(cst[,a1:a2]), col='gray70', border='gray30')

}

}

points(edd2$longitude, edd2$latitude,cex=2, pch=21,

col='black', bg=adjustcolor(mycols[colD], .6))

text(edd2$longitude, edd2$latitude, edd2$Site,

cex=0.7, pos=3, offset=0.6)

if(outPDF) dev.off()

#--------------------------------------------------------------

#------- FIGURE 4 ---------------------------------------------

# Maps of all sites by taxon

typeE <- liveE # choose liveE

typeD <- deadE # choose deadE

col.sea <- 'cyan1'

col.isl.out <- 'gray10'; col.isl.bg <- 'yellow1'

col.live <- 'black'; bg.live <- 'black'

col.dead <- 'black'; bg.dead <- 'black'

col.empty <- 'darkgray'; bg.empty <- 'white'

if(outPDF) pdf("FIGURE_TAXA_MAPS.pdf", height=6, width=4.5)

temppar <- par(mfcol=c(6,2), mar=c(0,0,0,0), oma=c(4,6,0.5,0.5))

for (j in 1:6) {

plot(edd2$longitude, edd2$latitude, xlab='', ylab='', axes=F,

cex.axis=0.8, ylim=c(24.65,24.87), type='n')

rect(-120,20,80,40, col=col.sea)

axis(2, las=1)

mtext(side=3, line=-1.3, adj=0.015, LETTERS[j], cex=0.8)

for (i in 2:length(plgs)) {

if (i < length(plgs)) {

a1 <- plgs[i]+1; a2 <- plgs[i+1]-1

polygon(t(cst[,a1:a2]), col=col.isl.bg, border=col.isl.out, lwd=0.5)

}

}

col.cd <- which(typeE2[,j]>0)

points(edd2$longitude[-col.cd], edd2$latitude[-col.cd],

cex=1, pch=21, col=col.empty, bg=bg.empty)

points(edd2$longitude[col.cd], edd2$latitude[col.cd], pch=21,

col=col.live, bg=adjustcolor(bg.live, .4), cex=1 + typeE2[,j][col.cd])

mtext(side=3, line=-1.1, adj=0.15, sort(tx.labels)[j],

font=3, cex=0.6)

if (j > 5) axis(1, at=seq(-80.95, -80.75, 0.1),

labels=seq(-80.95, -80.75, 0.1))

box()

}

for (j in 1:6) {

plot(edd2$longitude, edd2$latitude, xlab='', ylab='', axes=F,

cex.axis=0.8, ylim=c(24.65,24.87), type='n')

rect(-120,20,80,40, col=col.sea)

mtext(side=3, line=-1.3, adj=0.015, LETTERS[j+6], cex=0.8)

for (i in 2:length(plgs)) {

if (i < length(plgs)) {

a1 <- plgs[i]+1; a2 <- plgs[i+1]-1

polygon(t(cst[,a1:a2]), col=col.isl.bg, border=col.isl.out, lwd=0.5)}}

col.cd <- which(typeD2[,j]>0)

points(edd2$longitude[-col.cd], edd2$latitude[-col.cd], cex=1, pch=21,

col=col.empty, bg=bg.empty)

points(edd2$longitude[col.cd], edd2$latitude[col.cd], pch=21,

col=col.dead, bg=adjustcolor(bg.dead, .4), cex=1 + typeD2[,j][col.cd])

if (j > 5) axis(1, at=seq(-80.95, -80.75, 0.1),

labels=seq(-80.95, -80.75, 0.1))

mtext(side=3, line=-1.1, adj=0.15, sort(tx.labels)[j],

font=3, cex=0.6)

box()

}

mtext(outer=T, side=1, line=2.5, 'longitude', xpd=NA)

mtext(outer=T, side=2, line=3.5, 'latitude')

if(outPDF) dev.off()

par(temppar)

#--------------------------------------------------------------

#--------------------------------------------------------------

# BASIC STATS

paste('number of sites with live =',

sum(rowSums(liveE)>0))

paste('number of sites with dead =',

sum(rowSums(deadE)>0))

paste('number of sites with either =',

sum(rowSums(deadE+liveE)>0))

paste('number of sites with both =',

sum(rowSums(deadE*liveE)>0))

paste('number of sites with neither =',

sum(rowSums(deadE+liveE) == 0))

#--------------------------------------------------------------
